# Association of p16 as Prognostic Factors for Oropharyngeal Cancer: Evaluation of p16 in 1470 Patients for a 16 Year Study in Northeast China

**DOI:** 10.1155/2018/9594568

**Published:** 2018-09-17

**Authors:** Hong-xue Meng, Su-sheng Miao, Kexin Chen, Hui-ning Li, Guodong Yao, Jiashi Geng, Hongmei Wang, Qing-tao Shi, Jing He, Xionghui Mao, Fang-jia Tong, Lan-Lan Wei, Ji Sun, Dongfeng Tan, Qi You, Xiaomei Li, Jing-shu Geng

**Affiliations:** ^1^Department of Pathology, Harbin Medical University Cancer Hospital, Harbin, China; ^2^Department of Otolaryngology, Head and Neck Surgery, Harbin Medical University Cancer Hospital, Harbin, China; ^3^Department of Pathology, The First Affiliated Hospital of Heilongjiang University of Chinese Medicine, Harbin, China; ^4^Department of pathology, Harbin Medical University, Harbin, China; ^5^Department of Radiology, Harbin Medical University Cancer Hospital, Harbin, China; ^6^Department of Microbiology, Harbin Medical University, Harbin, China; ^7^Department of Gastroenterology, Harbin Medical University Cancer Hospital, Harbin, China

## Abstract

Human papillomavirus (HPV) is an etiological risk factor for oropharyngeal squamous cell carcinomas (OPSCC). Our study investigates the prevalence, prognostic, and clinicopathologic features of HPV-related oropharyngeal cancer in Northeast China and elucidates the involvement of p16 in the tumorigenesis and progression of OPSCC. Specimens from 1470 OPSCC patients collected from 2000 to 2016 were analyzed using the status of HPV by polymerase chain reaction (PCR) and p16 immunohistochemistry. Overexpression of p16 was observed in 81 (5.51%) of the 1470 cases, and HPV positive was present in 78 cases (5.31%) of the 1470 cases. HPV positive and p16 overexpression have a good concordance. However, we found that the etiological fraction of HPV in cancers of the OPSCCs was obviously lower in Northeast China than other cohorts previously reported. Interestingly, nearly 89% of patients with p16 expression were smokers, and nearly 70% of patients with p16 expression had a history of alcohol. Our study also demonstrates that p16 expression is significantly associated with early stage primary OPSCCs and the patients with p16 expression tend to show better survival following surgery and radiotherapy.

## 1. Introduction

Head and neck squamous cell carcinoma (HNSCC) has been defined as the sixth leading cause of cancer in the world [1]. High recurrence rates and nodal metastases always lead to high mortality of HNSCC. Especially, 5-year survival rates of HNSCC patients with cervical lymph node metastases are reduced by approximately 50% [2]. Conventionally, patients diagnosed with early stage HNSCC would have good prognosis after surgery and adjuvant radiation [3, 4].

Before HPV positive as a new risk factor for HNSCC was found, many risk factors had been reported, including tobacco, poor oral hygiene, and alcohol [5, 6]. Then the prevalence of HPV-related HNSCC, especially oropharyngeal squamous cell carcinomas (OPSCC), was largely observed in many populations in Western Europe, United States, and Australia [3–7]. Nonetheless, the prevalence, prognostic, significance, and correlations of high-risk HPV infection in OPSCCs in China cohort, accounting for 1/4 of the global population, remain blurry. And the precise pathogenesis and clinic pathologic features of HPV-related oropharyngeal cancer in Northeast China are still unclear.

High-risk human papillomavirus (HPV) infection causes the increase of OPSCC [8]. Many studies have shown that the prevalence of HPV-related OPSCC has been evaluated to range from 45 to 90% [3–7]. Moreover, a dominant subtype of HPV16 is thought to represent 90% of HPV-related OPSCC. HPV-related OPSCC is identified as a unique clinical entity. Patients with HPV associated SCC are expected to have the improved survival. Thus the clinical value of exploring the role of HPV in OPSCC is also beneficial to decrease treatment related side-effect [8].

p16 protein expression has been reported to be related to HPV infection, and p16 may be used as a predictive biomarker for HPV high-risk tumors [9]. p16 as a cyclin-dependent kinase inhibitor played an important role in inhibiting CDK4 and cyclin D1 complex dependent phosphorylation of Rb (retinoblastoma), as a tumor suppressor protein [10]. Viral oncoproteins E7 is always expressed in HPV-related cancers. Studies had shown an inhibitory effect of E7 on Rb activation by HPV infection [11, 12]. And inactivation of Rb by HPV-expressed E7 induced the transcription of the cyclin-dependent kinase inhibitor p16 [13]. Importantly, the expression of p16 was a positive indicator for improved survival. Several researches have demonstrated that p16 was a more effective independent prognostic factor for overall survival and progression-free survival than HPV status prediction [14, 15]. However, whether p16 immunohistochemistry could be used as a strong discriminator of clinical outcome in patients with OPSCC has not been defined. Larger studies are necessary to determine whether p16 can be used as well established prognostic variables, including T category, depth of invasion, and nodal status of OPSCC.

In our study, we first investigated the prevalence and prognostic and clinicopathologic features of HPV-related oropharyngeal cancer in Northeast China. Furthermore, we observed that p16 expression was significantly associated with early stage primary OPSCCs and that patients with p16 expression tend to show better survival following surgery and radiotherapy. Our results suggest that p16 may be a prognostic factor of OPSCCs in China.

## 2. Methods

### 2.1. Patients

This study enrolled 1470 patients with pathology-proven oropharyngeal cancer. Patients were recruited from Harbin Medical University Cancer Hospital (Cancer Center for Northeast China, Harbin, China) from January 2000 to February 2016. Tissues were obtained from patients during surgery.

### 2.2. Ethics Statement

According to the principles of the Declaration of Helsinki, we conducted this research. All participants in this study signed the written informed consent. The study had been approved by the Institutional Ethics Committee of Harbin Medical University Cancer Hospital.

### 2.3. Clinical Parameters

The clinical data of controls and IgAN patients, including age, history of smoking, gender, history of alcohol, and treatment, were collected.

### 2.4. Histopathological Diagnosis

All cases were diagnosed and categorized according to the WHO classification. All slides were reviewed by two pathologists and scored the pathological variables. International Collaboration on Oropharyngeal Cancer Network for Staging (ICON-S) has developed a TNM classification specific to HPV positive oropharyngeal cancer [16, 17]. We followed the TNM stage from 7th edition of the UICC/AJCC TNM classification: no lymph nodes as ICON-S N0; ipsilateral lymph nodes as ICON-S N1; bilateral or contralateral lymph nodes as ICON-S N2; lymph nodes larger than 6 cm as ICON-S N3, which resembles the N classification of nasopharyngeal carcinoma except the lack of a lower neck lymph node variable. The proposed ICON-S classification is as follows: stage I is T1-T2N0-N1, stage II is T1-T2N2 or T3N0–N2, and stage III is T4 or N3. Metastatic disease (M1) is classified as ICON-S stage IV.

### 2.5. Antibodies and Immunohistochemistry (IHC)

Formalin-fixed samples and paraffin-embedded sections (4 *μ*m thick) were first blocked with 1% H_2_O_2_. Then the samples were treated by antigen retrieval in trypsin for 30 min at 37°C, followed by immersion in citrate buffer (pH 6.0; Mitsubishi Chemical Medience, Tokyo, Japan) for 20 min at 120°C in an autoclave. Protein Blocking Agent (Streptavidin-Biotin Universal Detection System; Beckman Coulter, Marseille, France) was used to block the sections. And then the sections were incubated with the following primary antibodies overnight at 4°C: rabbit anti-human P16 (1 : 100, INK4a, IgG, Zhongshan, China). After that, sections were incubated with secondary antibodies from the Streptavidin-Biotin Universal Detection System (Beckman Coulter) and visualized by DAB. The negative controls were specific isotype control antibodies and phosphate-buffered saline (PBS; omitting primary antibodies).

For calculating the p16 [INK4a] expression, nuclear and cytoplasmic positivity were identified as positive reactions and were scored semiquantitatively as described by previous study [15]: negative score was <1% of positive cells; sporadic score was that isolated cells were positive but <5%; focal score was small cell clusters but <80% of positive cells; and diffuse score was >80% of positive cells. Positive cells with p16 expression were defined as strong and diffuse nuclear and cytoplasmic staining in at least 80 percent or more of the tumor cells.

### 2.6. DNA Extraction and PCR Analysis

Total DNA was extracted and purified from formalin-fixed, paraffin-embedded tissues by DNeasy Micro kit (Qiagen, Hilden, Germany). The resulting DNA was amplified for 35 cycles by PCR. The forward and reverse primers were listed as follows: *β*-globin 5′-GAA GAG CCA AGG ACA GGT AC-3′ (forward) and 5′-CAA CTT CAT CCA CGT TCA CC-3′ (reverse); HPV 5′-CGT CCM ARR GGA WAC TGA TC-3′ (forward) and 5′-GCM CAG GGW CAT AAY AAT GG-3′ (reverse).

### 2.7. Statistical Analysis

Student's *t*-test was performed to estimate the significant difference between HPV positive OPSCC patients and HPV negative OPSCC patients. We also analyzed the correlation among clinical presentation, HPV state, and P16 by Spearman's correlation analysis and Pearson's correlation analysis (SAS Institute Inc., Cary, NC, USA). *P* values less than 0.05 were considered as significant differences.

## 3. Results

### 3.1. Clinical and Pathological Parameters

The clinicopathological characters of the 1470 cases of OPSCC were represented in Table 1. Most of patients were males (*n* = 1167; 79.39%) and smokers (*n* = 1296; 88.16%). About sixty percent (60.54%) of 890 patients were alcohol consumers. Among all these patients, patients with surgery only were 1257 cases, patients with surgery followed by radiotherapy were 205 cases, patients with surgery followed by chemotherapy were 5 cases, and patients with surgery followed by radiotherapy and chemotherapy were 3 cases. All of the patients had available follow-up information. 286 patients (19.46%) presented with residual disease, but 1184 patients (80.54%) initially obtained a complete response (CR) after finishing the initial CRT. 62.75% (743/1184) of patients maintained the CR during follow-up; however, 37.24% (441/1184) of patients subsequently showed recurrence or metastasis.

According to the histologic typing, 174 (11.84%) of the 1470 cases were SCC NOS/conventional nonkeratinizing and 1271 (86.46%) cases were conventional keratinizing. For the differentiation, 467 (31.77%) cases were well, 818 (55.65%) cases were moderate, and 185 (12.95%) cases were poor. Most cases (615, 41.83%) have lymphovascular invasion. For TNM stage statistic, 1395 patients had low–T stage (T1/T2) OPSCC tumors and 75 patients had high–T stage (T3/T4) OPSCC tumors. Moreover, patients with clinically positive lymph node metastasis (N+) were 615 (41.84%). For clinical stage statistic, patients with low clinical stage (I/II) were 91.02% (1338/1470) and high clinical stage (III/IV) were 8.98% (132/1470) (Table 2).

### 3.2. The Relationship between p16 Protein Overexpression and HPV Status

**Figure 1:**
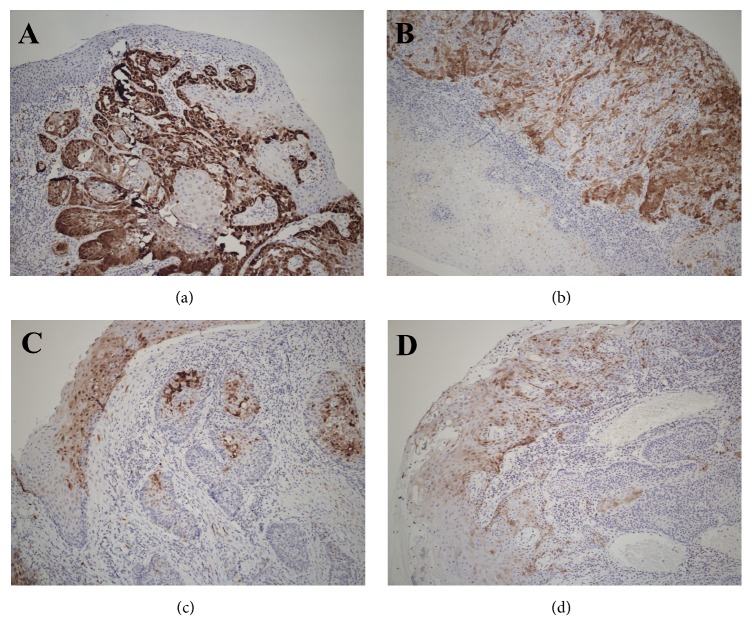
*Expression of P16-positive cells in oropharyngeal cancer*. Immunohistochemical analysis was used to show the expression of P16-positive cells in oropharyngeal cancer (magnification: ×400); nuclear and cytoplasmic positivity were classified as positive reactions and were scored as (a) diffuse (>80% of the cells were stained); (b) focal (small cell clusters, but <80% of the cells were positive); (c) sporadic (isolated cells were positive but <5%); (d) negative (<1% of cells were positive).

**Figure 2:**
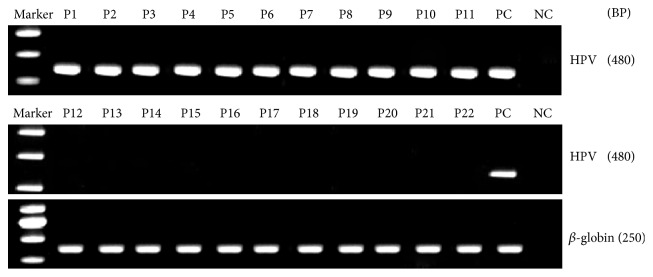
*HPV DNA-PCR of oropharyngeal cancer.* Using PCR, HPV state was detected in Oropharyngeal cancer patients.

5.51% (81/1470) of OPSCC samples were detected to p16 overexpression by immunohistochemistry (Figure 1). HPV was positive in 78 cases (5.31%) of the 1470 cases by PCR (Figure 2).

Good concordance between HPV positive status and p16 overexpression was established, which was with high sensitivity (100%) and high specificity (96%; Table 3). Consistently, we found that HPV status was significantly more frequently present among the young (age: 46–55) (*P* < 0.01), males (*P* < 0.05), conventional keratinizing type (*P* < 0.01), moderate and poor differentiation (*P* < 0.05), low T stage (*P* < 0.05), lymph node metastasis (*P* < 0.05), high clinical stage (*P* < 0.05), and p16 overexpression (*P* < 0.01) cases (Tables 3 and 4). Specifically, HPV and p16 positive patients usually maintained the CR more during follow-up. Our results also indicated that p16 expression may be a prognostic marker with an improved response to both radiation therapy and chemotherapy.

### 3.3. p16 Expression Was Significantly Associated with Improved Survival

Among all 1470 cases, positive p16 expression was linked with markedly improved overall survival (OS, *P* = 0.05), but this result was not significant in multivariate analysis (Table 5).

## 4. Discussion

This study including 1470 patients over a period of 16 years has a few inherent biases typical of a retrospective cohort. The principles of surgical management and patient selection for radiotherapy have essentially remained the same during this period, although there has been increasing use of adjuvant chemotherapy and highly conformal radiation techniques. Consequently, we have used HPV status detection as the gold standard to evaluate the potential clinical value of other prognostic markers for HPV-related OPSCC.

The percentage of HPV positive OPSCC in northeast Chinese patients calculated by our research was substantially lower than that published by recent meta-analysis data in oropharyngeal cancers [18]. The percentage of HPV positive OPSCC was 11.7% in an Eastern Chinese population and 21.7% in Southern Chinese patients as previously reported [19, 20]. The discrepancy may be related to the differences in the geographic origin of patients, heterogeneous laboratory procedures, or different methods used to detect HPV status.

We also found that HPV status was significantly correlated to sex and age of patients. About sex, HPV positive estimates were substantially higher in men than in women in our cohort, different from European cohort in other studies [3–7]. Additionally, we also found that HPV positive estimates were substantially higher in 46–55 ages. Finally, the incidence of tobacco smoking was 88.16%  (*n* = 1296) in our cohort and nearly 89% of patients with p16 expression were smokers. Sixty (60.54%) of 890 patients were alcohol consumers, and nearly 70% of patients with p16 expression had a history of alcohol. The carcinogenic effects of smoking and alcohol mediated through p53 mutations are notable.

In our results, we observed that OPSCC patients with p16 overexpression had significantly longer disease-specific survival than p16 negative patients following surgery as well as postoperative adjuvant radiotherapy, which was consistence with published data about the potential prognostic maker of p16 in oropharyngeal cancer. It suggested that p16 may be a biomarker for predicting the prognosis of OPSCC patients in China.

HPV and p16 as biomarkers or therapeutic targets in the treatment of HNSCC have the growing consensus of the importance [21]. In our study, p16 positive patients had significantly longer disease-specific survival on univariable analysis, which was essentially equivalent to that published by previous reports [22–24]. However, p16 expression was not only an independent predictor of survival on multivariable analysis. As discussed above, it may be reasonable to assume that p16 expression may also mediate survival of OPSCC patients by controlling the proliferative capacity and invasive potential of the primary tumor.

## 5. Conclusion

Our study demonstrates that p16 expression is significantly associated with early stage primary OPSCCs and that patients with p16 expression tend to show better survival following surgery and radiotherapy. p16 expression, as well as HPV status, may be a prognostic maker of OPSCCs in China. Furthermore, the etiological fraction of HPV in cancers of the OPSCCs is substantially lower in Northeast China than that in United States and Western Europe. Thus, the real prevalence of HPV in OPSCCs is still the future burden. Further researches will define the more detailed mechanisms underlying HPV involvement in OPSCCs.

## Figures and Tables

**Table 1:** Profiles and clinical parameters of patients.

Clinicopathological findings		
Variable	*n*	%
Age at diagnosis, years		
⩽45	85	5.78
46–55	469	31.9
56–65	624	42.45
*⩾*66	292	19.86
Mean (SD)	58.24 ± 6.64	
Age range	31–86	
Sex		
Male	1167	79.39
Female	303	20.61
History of smoking		
Yes (current/former)	1296	88.16
No (never)	174	11.84
History of alcohol		
Yes (current/former)	890	60.54
No (never)	580	39.46
Treatment		
Surgery alone	1257	85.51
Surgery + radiotherapy	205	13.95
Surgery + chemoradiotherapy	5	0.34
Surgery + radiotherapy + chemoradiotherapy	3	0.2
Event after initial CRT		
Residual tumor (PD, SD, and PR)	286	19.46
CR followed by recurrence/metastasis	441	30
Durable CR	743	50.54

Continuous variables are given as mean ± standard deviation.

**Table 2:** Histological diagnosis of patients.

Histological diagnosis	*n*	%
Squamous cell carcinoma		
SCC NOS/conventional nonkeratinizing	174	11.84
Conventional keratinizing	1271	86.46
Conventional exophytic keratinizing	14	0.95
Basaloid/papillary	2	0.14
Verrucous	1	0.07
Sarcomatoid	4	0.27
Undifferentiated carcinoma	2	0.14
Adenosquamous carcinoma	2	0.14
Differentiation		
Well	467	31.77
Moderate	818	55.65
Poor	185	12.59
Lymphovascular invasion	615	41.83
Perineural invasion	441	30
Extracapsular spread	244	16.6
Bone invasion	148	10.07
Pathological T category		
T1	182	12.38
T2	1213	82.52
T3	61	4.15
T4	14	0.95
Pathological N category		
N0	855	58.16
N1	422	28.71
N2a	119	8.1
N2b	57	3.88
N2c	17	1.16
N3	0	
Clinical stage		
I/II	1338	91.02
III/IV	132	8.98

**Table 3:** The relationship between p16 overexpression, HPV status, and clinical parameters of patients.

Variable	p16-IHC		*P*	HPV DNA-PCR		*P*
	*n* = 1470			*n* = 1470		
	Positive (*n* = 81)	Negative (*n* = 1389)		Positive (*n* = 78)	Negative (*n* = 1392)	
*Patient characteristics*						
Age at diagnosis, years						
⩽45	12	73	0.0034	11	74	0.0012
46–55	43	426	0.00004	41	428	0.000058
56–65	16	608	0.00002	16	608	0.000056
*⩾*66	10	282	0.081	10	282	0.109
Sex						
Male	72	1095	0.029	69	1098	0.0417
Female	9	294	0.041	9	294	0.041
History of smoking						
Yes (current/former)	72	1224	0.835	69	1227	0.933
No (never)	9	165	0.833	9	165	0.833
History of alcohol						
Yes (current/former)	57	833	0.062	54	836	0.106
No (never)	24	556	0.082	24	556	0.08
*Treatment*						
Surgery alone	57	1200	0.00006	55	1202	0.00011
Surgery + radiotherapy	24	181	0.00003	23	182	0.000047
Surgery + chemoradiotherapy	0	8	0.49	0	8	0.501
*Event after initial CRT*						
Residual tumor (PD, SD, and PR)	21	265	0.13	21	265	0.086
CR followed by recurrence/metastasis	35	406	0.007	33	408	0.014
Durable CR	25	718	0.0003	24	719	0.00033

**Table 4:** The relationship between p16 overexpression, HPV status, and Histological diagnosis.

Variable	p16-IHC		*P*	HPV DNA-PCR		*P*
	*n* = 1470			*n* = 1470		
	Positive (*n* = 81)	Negative (*n* = 1389)		Positive (*n* = 78)	Negative (*n* = 1392)	
*Histological diagnosis*						
Squamous cell carcinoma						
SCC NOS/conventional nonkeratinizing	13	161	0.862	12	162	0.318
Conventional keratinizing	66	1205	0	64	1207	0.242
Conventional exophytic keratinizing	2	12	0.169	2	6	0.127
Basaloid/papillary	0	2	0.732	0	2	0.737
Verrucous	0	1	0.809	0	1	0.813
Sarcomatoid	0	4	0.628	0	4	0.635
Undifferentiated carcinoma	0	2	0.732	0	2	0.737
Adenosquamous carcinoma	0	2	0.732	0	2	0.737
Differentiation						
Well	24	443	0.0017	23	444	0.656
Moderate	20	798	0	19	799	0
Poor	37	148	0.000002	36	149	0
Lymphovascular invasion	56	559	0.0027	56	559	0
Perineural invasion	77	364	0.036	76	365	0
Extracapsular spread	10	234	0.068	8	236	0.122
Bone invasion	4	144	0	4	144	0.136
Pathological T category						
T1	4	178	0.073	4	178	0.0456
T2	73	1140	0	70	1143	0.084
T3	2	59	0.394	2	59	0.471
T4	1	13	0	1	13	0.758
Pathological N category						
N0	26	829	0.806	24	831	0
N1	25	397	0.02	24	398	0.679
N2a	19	100	0.00066	19	100	0
N2b	9	48	0.00052	9	48	0.00031
N2c	2	15	0.255	2	15	0.232
N3	0	0		0	0	
*Clinical stage*						
I/II	6	126	0.611	6	126	0.683
III/IV	75	1263	0.611	72	1266	0.683

**Table 5:** Association between p16 status and survival.

	Univariable (*p* value)	Multivariable, HR (95% CI)	In patients receiving radiotherapy
		*P* value	(*P* value)
Disease-specific	0.03^a^	0.19 (0.02–1.38)	0.14^a^
		0.100^b^	
Disease-free	0.14^a^	0.67 (0.32–1.36)	0.014^a^
		0.266	
Overall survival	0.05^a^	0.44 (0.15–1.23)	0.038^a^
		0.118	

^a^Log rank test *P* value; ^b^adjusting for effect of depth of invasion alone.
